# Technical considerations for surgical explantation of microaxial, catheter-based percutaneous ventricular assist device via the ascending aorta

**DOI:** 10.1016/j.xjtc.2025.08.018

**Published:** 2025-09-05

**Authors:** Ryosuke Marushima, Shintaroh Koizumi, Hiroki Kohno, Goro Matsumiya

**Affiliations:** Department of Cardiovascular Surgery, Chiba University Hospital, Chiba, Japan

**Figure:**
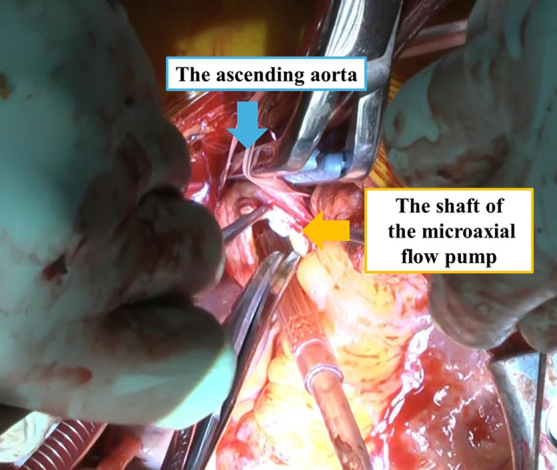
Transecting the shaft of a microaxial flow pump via the AAo.

**Central Message**

We report the successful explantation of a microaxial, catheter-based percutaneous ventricular assist device via the AAo during durable LVAD implantation.

The Impella 5.5 (Abiomed Inc) is a microaxial, catheter-based percutaneous ventricular device for temporary mechanical circulatory support used in the management of cardiogenic shock. It also serves as a bridge to decision regarding further treatments such as durable left ventricular assist device (LVAD) or heart transplant. The Impella 5.5 is typically inserted via the axillary artery (AxA) and is generally pulled out through the original insertion site. However, in certain cases, vascular or anatomic challenges complicate the device explantation.

This report describes a 33-year-old woman diagnosed with idiopathic dilated cardiomyopathy who was supported with the Impella 5.5 via the right AxA. Because of the risk of vascular complications, the device was explanted directly from the ascending aorta (AAo) during the durable LVAD implantation.

## Case

A 33-year-old woman at 28 weeks of gestation, with a family history of idiopathic dilated cardiomyopathy, was admitted to a local hospital with decompensated heart failure. As her condition progressively worsened, she was transferred to our hospital for consideration of mechanical circulatory support. Initial support was provided with venoarterial extracorporeal membrane oxygenation and an Impella CP (Abiomed Inc) to stabilize her hemodynamics. Despite this, her condition continued to deteriorate with worsening pulmonary congestion and multiorgan dysfunction. Therefore, the decision was made to escalate her mechanical circulatory support with an upgrade from the Impella CP to the Impella 5.5.

**Figure 1:**
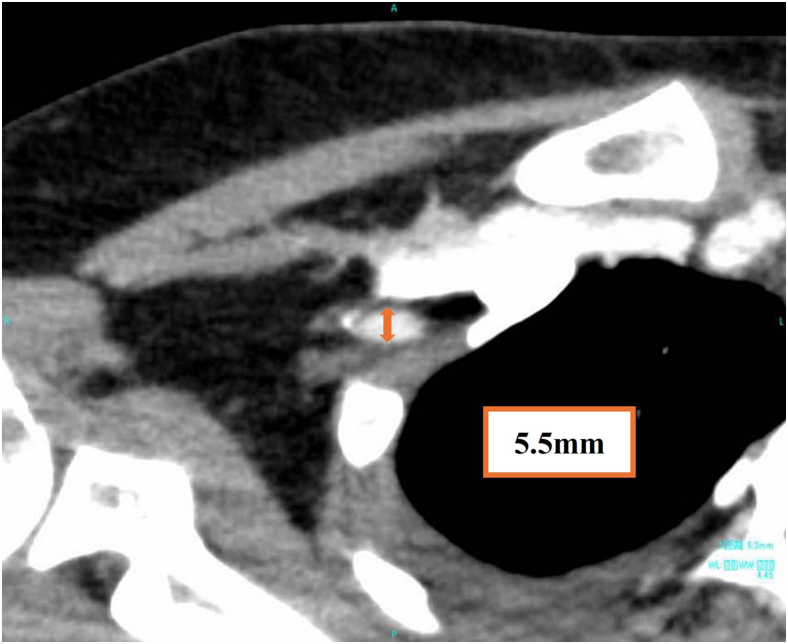
Computed tomography image of the right *AxA*. The minimal artery diameter was 5.5 mm at the first portion of the right *AxA*.

The access route for Impella 5.5 insertion via the bilateral AxA was evaluated using contrast-enhanced computed tomography. The minimal artery diameter of the access route via the right AxA was 5.5 mm, which was smaller than the manufacturer's recommended minimal artery diameter of 7.0 mm (Figure 1). In contrast, the access route via the left AxA was significantly smaller. An alternative approach with direct aortic insertion was discussed in a multidisciplinary meeting. However, it was ultimately decided to proceed with the insertion via the right AxA, given the uncertainty regarding the duration of Impella 5.5 support at this stage and the potential risk of mediastinal infection should durable LVAD support be required in the future.

Impella 5.5 insertion was performed using the standard technique under fluoroscopic and transesophageal echocardiography guidance. The second portion of the right AxA was surgically exposed, and a 10-mm prosthetic graft was anastomosed. Significant resistance was encountered during advancement of the Impella 5.5 through the proximal segment of the right AxA. By rotating the device with manual manipulation, it was successfully advanced through the segment and positioned in the left ventricle (LV) without any vascular complication.

**Figure 2:**
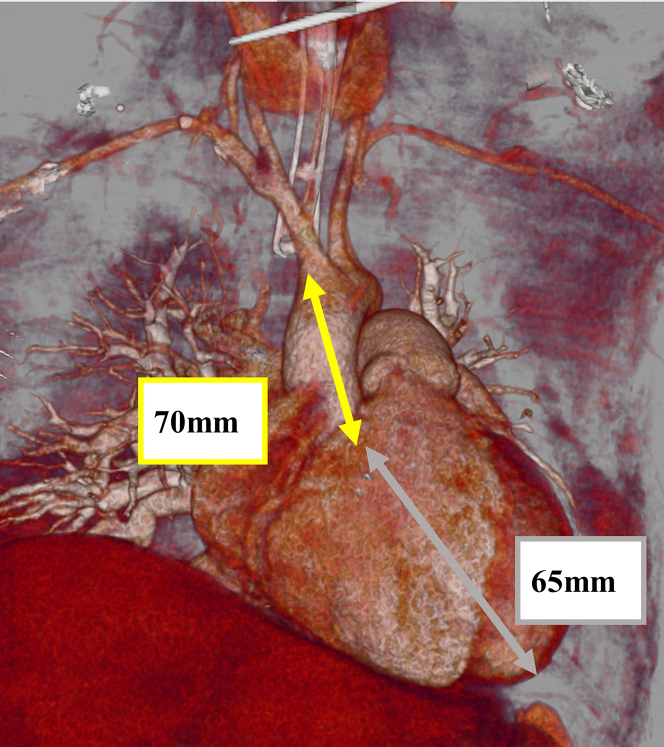
Three-dimensional reconstruction image of the access route and heart. The distance from the brachiocephalic artery bifurcation to the aortic valve was 70 mm (*yellow arrow*). The distance from the aortic valve to the LV apex was 65 mm (*gray arrow*).

After the upgrade to the Impella 5.5 device, her cardiac function gradually improved, allowing successful weaning from venoarterial extracorporeal membrane oxygenation. However, her cardiac function did not recover enough to wean from the Impella 5.5, and she was subsequently scheduled for durable LVAD implantation as long-term support. Given the technical and anatomic challenges during the previous Impella 5.5 insertion, explantation from the original insertion site was considered unpredictable and associated with a high risk of vascular and embolic complications. Therefore, our surgical team planned to explant the Impella 5.5 device directly via the AAo during durable LVAD implantation. Her computed tomography revealed 70 mm from the origin of the brachiocephalic artery to the aortic valve and 65 mm from the aortic valve to the LV apex (Figure 2). Given that the length of the Impella 5.5 pump portion was 125 mm, it was theoretically feasible to advance the Impella 5.5 toward the LV apex and then clamp both the AAo and the Impella shaft at the level of brachiocephalic artery bifurcation.

The operation was performed via median sternotomy. Cardiopulmonary bypass was initiated with aortic arch return and bicaval venous drainage, and the Impella flow was decreased. By using epi-aortic echocardiography, the location of the Impella 5.5 pump was identified in the AAo and it was advanced toward the LV apex carefully, ensuring that its shaft was located at the level of the aortic clamping site. The aortic crossclamp with a soft jaw was carefully applied across the AAo and itsshaft (Video 1). The heart was arrested with retrograde cardioplegia. During the administration of cardioplegia, a longitudinal aortotomy was made on the AAo, which was to become the LVAD outflow anastomosis site, and the junction between the pump and the shaft of the Impella 5.5 was exposed. The shaft was transected with Mayo scissors, and the pump was explanted through the same aortotomy. A piece of LVAD outflow graft was anastomosed to the aortotomy and clamped, and then the crossclamp was released. Durable LVAD implantation was completed in the standard fashion. The remaining portion of the Impella 5.5 was subsequently pulled out via the right AxA without complications after releasing the aortic clamp. The patient's postoperative course was uneventful, and she is currently awaiting heart transplant. According to our institutional policy, ethical approval was not required for this case report. Written informed consent for publication was obtained from the patient.

## Discussion

The Impella 5.5 has been established as a temporary ventricular assist device for cardiogenic shock. However, there are potential complications during its removal, such as embolization, vascular injury, brachial plexus injury, and device fracture. According to the manufacturer's recommendations, a minimum artery diameter of 7.0 mm is required for insertion of the Impella 5.5. However, with growing clinical experience, it has been inserted with smaller artery diameters in select cases.^1^, ^2^, ^3^ On the other hand, an explantation of the Impella 5.5 through the original insertion site may be technically challenging and unpredictable in patients with such a small artery diameter, potentially leading to vascular complications.

For patients with small artery diameter, several alternative approaches to insertion of the Impella 5.5, such as via the AAo or innominate artery, have been reported.^4^, ^5^, ^6^ These approaches can reduce the risk of vascular complications associated with the AxA approach. However, the AxA approach offers several advantages over these alternative approaches. First, the AxA approach is associated with improved patient comfort, which may facilitate early mobilization and rehabilitation. Second, it carries a lower risk of mediastinal infection, which is an important consideration, particularly in patients being scheduled for LVAD implantation or heart transplant. Therefore, Impella 5.5 insertion via the AxA remains the preferred approach, even in patients with small artery diameter.

Several considerations must be addressed when planning to explant the Impella 5.5 via the AAo during cardiac surgery. To explant the device safely, it is mandatory to clamp the shaft of the Impella 5.5 and transect it in the AAo. Given that the length of the pump portion of the Impella 5.5 is 125 mm, the distance from the brachiocephalic artery and LV apex must theoretically exceed 125 mm. In our case, the distance measured 135 mm, and simply advancing the Impella 5.5 toward the LV apex enabled successful explantation. However, even in this setting, intraoperative findings revealed that the tip of the Impella 5.5 was abutting the LV apex, indicating that the available length was marginally sufficient. For patients with a short AAo or a small LV cavity, the Impella direct aortic implantation technique should be selected as the initial approach. The use of epi-aortic ultrasound is essential for this procedure. As previously reported, epi-aortic ultrasound is highly effective for identifying the shaft of the Impella 5.5 and enabling safe clamping during Impella-supported cardiac surgery.^7^ In the context of explanting the Impella 5.5 via the AAo, it is also crucial to confirm there is sufficient shaft length in the AAo to allow for safe transection. Epi-aortic ultrasound provides precise localization of both the shaft and the pump portion of the Impella 5.5, thereby facilitating appropriate planning and execution of the explantation procedure.

## Conclusions

We reported the successful explantation of the Impella 5.5 device via the AAo during durable LVAD implantation. Although this technique has anatomic limitations, it offers an alternative and safe method for Impella 5.5 explantation in patients at high risk for vascular complications. This approach may broaden the applicability of axillary access for Impella 5.5 insertion, even in borderline anatomic situations.

## Conflict of Interest Statement

The authors reported no conflicts of interest.

The *Journal* policy requires editors and reviewers to disclose conflicts of interest and to decline handling or reviewing manuscripts for which they may have a conflict of interest. The editors and reviewers of this article have no conflicts of interest.

## Supplementary Data

Video 1 Surgical explantation of the Impella 5.5 via the AAo. The video illustrates the use of epi-aortic echocardiography for precise identification of the Impella shaft, crossclamping the AAo, and cutting the shaft via the AAo incision. Video available at: https://www.jtcvs.org/article/S2666-2507(25)00357-8/fulltext.
