# Supplementary figures and images for: Technical considerations for surgical explantation of microaxial, catheter-based percutaneous ventricular assist device via the ascending aorta
Source: JTCVS Tech. 2025 Sep 5;34:129–31. doi: 10.1016/j.xjtc.2025.08.018 (PMC12682964; doi:10.1016/j.xjtc.2025.08.018)

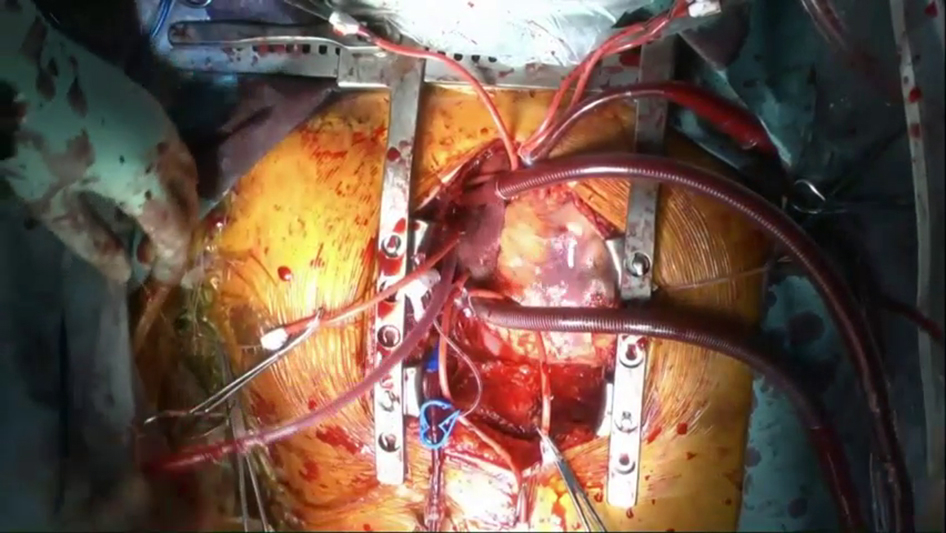

Supplement: Video 1 — Surgical explantation of the Impella 5.5 via the AAo. The video illustrates the use of epi-aortic echocardiography for precise identification of the Impella shaft, crossclamping the AAo, and cutting the shaft via the AAo incision. Video available at: https://www.jtcvs.org/article/S2666-2507(25)00357-8/fulltext. [file fx2.jpg]
